# RNA-Seq analysis of seasonal and individual variation in blood transcriptomes of healthy managed bottlenose dolphins

**DOI:** 10.1186/s12864-016-3020-8

**Published:** 2016-09-08

**Authors:** Jeanine S. Morey, Marion G. Neely, Denise Lunardi, Paul E. Anderson, Lori H. Schwacke, Michelle Campbell, Frances M. Van Dolah

**Affiliations:** 1 National Centers for Coastal Ocean Sciences, National Ocean Service, NOAA, 331 Fort Johnson Rd, Charleston, SC 29412 USA; 2 Department of Life Sciences and Biotechnology, University of Ferrara, via L. Borsari 46, 44121 Ferrara, Italy; 3 Department of Computer Science, College of Charleston, Charleston, SC 29424 USA; 4 Dolphin Quest, Waikoloa, Hawaii USA; 5 Present Address: Graduate Program in Marine Biology, University of Charleston, Charleston, SC 29412 USA

**Keywords:** *Tursiops truncatus*, Bottlenose dolphin, Blood transcriptome, RNA-seq, Globin-reduction

## Abstract

**Background:**

The blood transcriptome can reflect both systemic exposures and pathological changes in other organs of the body because immune cells recirculate through the blood, lymphoid tissues, and affected sites. In human and veterinary medicine, blood transcriptome analysis has been used successfully to identify markers of disease or pathological conditions, but can be confounded by large seasonal changes in expression. In comparison, the use of transcriptomic based analyses in wildlife has been limited. Here we report a longitudinal study of four managed bottlenose dolphins located in Waikoloa, Hawaii, serially sampled (approximately monthly) over the course of 1 year to establish baseline information on the content and variation of the dolphin blood transcriptome.

**Results:**

Illumina based RNA-seq analyses were carried out using both the Ensembl dolphin genome and a *de novo* blood transcriptome as guides. Overall, the blood transcriptome encompassed a wide array of cellular functions and processes and was relatively stable within and between animals over the course of 1 year. Principal components analysis revealed moderate clustering by sex associated with the variation among global gene expression profiles (PC1, 22 % of variance). Limited seasonal change was observed, with < 2.5 % of genes differentially expressed between winter and summer months (FDR < 0.05). Among the differentially expressed genes, cosinor analysis identified seasonal rhythmicity for the observed changes in blood gene expression, consistent with studies in humans. While the proportion of seasonally variant genes in these dolphins is much smaller than that reported in humans, the majority of those identified in dolphins were also shown to vary with season in humans. Gene co-expression network analysis identified several gene modules with significant correlation to age, sex, or hematological parameters.

**Conclusions:**

This longitudinal analysis of healthy managed dolphins establishes a preliminary baseline for blood transcriptome analysis in this species. Correlations with hematological parameters, distinct from muted seasonal effects, suggest that the otherwise relatively stable blood transcriptome may be a useful indicator of health and exposure. A robust database of gene expression in free-ranging and managed dolphins across seasons with known adverse health conditions or contaminant exposures will be needed to establish predictive gene expression profiles suitable for biomonitoring.

**Electronic supplementary material:**

The online version of this article (doi:10.1186/s12864-016-3020-8) contains supplementary material, which is available to authorized users.

## Background

High-throughput blood gene expression profiling has been broadly applied in human medicine for the identification of health status and disease, monitoring responses to drug therapies, defining disease prognosis, and identifying exposures to environmental toxicants [1, 2]. Blood plays a central role in physiological homeostasis and immunity; transporting nutrients, hormones, metabolites, and cytokines to all tissues in the body. Immune cells recirculate between the blood and lymphoid tissues and migrate to sites of injury or pathological insult, where genes responsive to specific exposures or disease states are induced. The blood transcriptome therefore has the capacity to reflect both systemic exposures and pathological changes in other organs of the body. In humans distinct blood transcriptomic signatures have been demonstrated for over 35 different medical conditions [2] and have been successfully employed to gain insight into processes and/or prognoses of cancer, heart disease, stroke, autoimmunity, neurological disorders, and responses to vaccines [1].

Blood transcriptome analysis has met similar success in veterinary medicine to identify markers of infectious disease and pathological conditions in economically relevant species (cow, pig, horse, sheep) and companion species (dog, cat) (for review see [1]). The application of high throughput transcriptomics to wildlife has to date been more limited, in part because of the lack of sequenced genomes for many species, as well as the difficulty associated with obtaining samples. In marine mammals, a microarray study in California sea lions identified blood gene expression profiles that distinguished between two prevalent disease states, domoic acid poisoning and leptospirosis [3], while qPCR of selected genes expressed in blood from California sea lions identified signatures of polycyclic aromatic hydrocarbons (PAH) exposures associated with wildfires [4]. Transcriptomic analysis of peripheral blood identified potential markers of nutritional stress in Steller sea lions [5]. Microarray based blood gene expression profiles in bottlenose dolphins identified stress responses associated with handling during capture-release studies [6] and were able to classify animals according to polychlorinated biphenyl (PCB) exposure levels [7].

Mammalian blood is composed of approximately 55 % plasma and 45 % cellular material. The cellular components are made up of 96 % red blood cells and 4 % leukocytes, which can vary in composition over time, and is thus one of the most dynamic tissues in the body. Because the gene expression profiles of leukocyte classes differ significantly, changing cellular composition can significantly alter the global blood transcriptome. Further development of blood transcriptomics as a tool for identifying signatures indicative of disease, exposures, or health status in wildlife requires knowledge of both the natural intra- and inter-animal variation in gene expression. For example, longitudinal studies of gene expression in human blood have found minimal intra-individual variation over the course of 1 month, but significant variability was observed by 3 to 6 months and this baseline variation must be taken into account [8, 9]. Recent studies suggest that seasonal patterns in human behavior, physiology, and disease susceptibility may be related to underlying fluctuations in hematological parameters, as blood cell composition shows annual variation [10]. Studies on ethnically and geographically diverse populations identified significant seasonal variation in over 4000 blood gene transcripts that are biologically and clinically relevant [11]. Circadian clock genes responsive to changes in day length are among the seasonally expressed genes, as well immune functions suggestive of a proinflammatory status during the winter months among populations living at high latitudes. In contrast, seasonal trends in gene expression from tropical populations correlate with rainy season during which individuals experience higher exposure to infectious agents [11]. Many of the observed differences reflect seasonal changes in cellular composition of the blood [10, 11].

Here we sought to establish baseline data on blood transcriptomes in bottlenose dolphins, *Tursiops truncatus*. Four healthy, managed dolphins located in Waikoloa, Hawaii, were sampled approximately monthly for the duration of 1 year. Hematological parameters including blood cell counts and serum chemistry were measured quarterly. Transcriptomic analysis of blood using RNA sequencing can be a challenge because globin transcripts are highly abundant, up to 76 % in human blood, 46 % in porcine blood [12], potentially limiting the detection and coverage of other transcripts of interest or incurring additional cost of deeper sequencing to obtain adequate transcriptome sampling. In preliminary analyses we found that bottlenose dolphin blood was dominated by globin transcripts and therefore developed a globin depletion protocol specific for bottlenose dolphin.

Because a fully annotated bottlenose dolphin genome is currently lacking, we conducted parallel analyses of the dolphin blood transcriptome, one assembled using the Ensembl dolphin genome (2.59X coverage) as a guide and the other assembled *de novo* using Trinity. Seasonal differences in gene expression were observed and patterns of gene expression over the sampling year were assessed for rhythmicity. In addition, network analysis identified several co-expressed gene modules with correlation to clinical parameters. The observed correlation of gene co-expression modules with clinical measurements suggests that blood transcriptomics may be informative of health status and disease in bottlenose dolphins, once a larger database of blood transcriptomes is established.

## Methods

### Animals, experimental design and sample collection

Blood samples were collected in PAXgene (Qiagen, Valencia, CA) tubes from the ventral side of the flukes of four managed *T. truncatus* residing at Dolphin Quest, Waikoloa, Hawaii at approximately monthly intervals during 2013. All research was approved by the Dolphin Quest Research Committee and carried out according to standards and guidelines of the AMMPA (Alliance of Marine Mammal Parks and Aquariums). The dolphins sampled for this study included two males, ages 5 (Hua, *n* = 8) and 17 (Kainalu, *n* = 7), and two females, ages 12 (Keo, *n* = 9) and 28 (Pele, *n* = 7). All animals are trained to participate in monthly veterinary checkups including routine blood draws, which were conducted in the mornings after overnight fasting. Hematological parameters were measured quarterly on samples collected in parallel with the transcriptome samples. Only samples from healthy animals were used; defined as bright, alert, responsive (BAR) animals demonstrating baseline behavior and appetite and blood chemistry within normal ranges. All samples and associated physical and hematological parameters collected are listed in Table 1 and Additional file 1: Table S1. Blood tubes were stored at −80 °C until extracted for RNA.

**Table 1:** All globin depleted samples and associated physical parameters measured

Animal	Sex	Age (years)	Sample Date	Monthly Mean Water Temp (°C)	Daylength (hours)
Hua	male	5	2/6/13	24.6	11.36
			3/21/13	24.4	11.78
			4/27/13	24.8	12.78
			5/23/13	25.2	13.15
			7/8/13	25.9	13.25
			8/23/13	26.4	12.65
			10/11/13	26.4	11.75
			12/2/13	25.1	11
Kainalu	male	17	2/5/13	24.6	11.36
			4/14/13	24.8	12.58
			5/25/13	25.2	13.18
			6/15/13	25.6	13.3
			7/8/13	25.9	13.25
			9/13/13	26.6	12.27
			12/2/13	25.1	11
Keo	female	12	2/13/13	24.6	11.48
			3/8/13	24.4	11.88
			4/1/13	24.8	12.3
			5/7/13	25.2	12.95
			6/12/13	25.9	13.3
			8/28/13	26.4	12.57
			10/29/13	26.4	11.45
			11/27/13	25.9	11.05
			12/9/13	25.1	11
Pele	female	28	2/13/13	25.1	11
			3/21/13	24.4	12.13
			5/24/13	25.2	13.16
			7/13/13	25.9	13
			8/22/13	26.4	12.67
			9/7/13	26.6	12.37
			12/13/13	25.1	10.95

### RNA extraction

Whole blood RNA was extracted using a PAXgene Blood RNA Kit (Qiagen, Valencia, CA), according to the manufacturer’s protocol with on-column DNase digestion to remove contaminating DNA. RNA concentrations were evaluated using a NanoDrop spectrophotometer (Thermo Fisher Scientific, Wilmington, DE) and RNA quality was assessed using an Agilent Bioanalyzer 2100 (Agilent Technologies, Inc., Santa Clara, CA). Only samples with a RIN (RNA Integrity Number) ≥ 7 were sequenced.

### Hemoglobin depletion

RNA-seq analysis of a test sample revealed a high percentage of globin transcripts in the dolphin peripheral blood transcriptome (65–75 % of reads). Therefore a modified Affymetrix globin depletion protocol utilizing RNase H was performed [12–14] in order to improve the diversity of transcripts detected by RNA-seq. Briefly, 500 or 1000 ng total RNA was hybridized with 2 μM each of 2 HBA oligonucleotides (5’-GGTATTTGGAGGTCAGCACGG-3’ and 5’-ATGGACCGAGGGCGTGAAAT-3’) and 1 μM each of HBB (5’-CTGAAGCTCCGGGGTGAATTC-3’) and HBM (5’-GTCAGGAACTTATCCCACACCAC-3’) oligonucleotides in hybridization buffer (100 mM Tris-HCl, pH 7.6, 200 mM KCl) at 70 ° C for 5 m and cooled to 4 ° C. The RNA-DNA hybrids were then digested with 1 U RNase H (Ambion, Thermo Fisher Scientific, Wilmington, DE) in 100 mM Tris-HCl, pH 7.6, 20 mM MgCl_2_, 0.1 mM DTT, and 40 U SUPERase-In (Ambion, Thermo Fisher Scientific, Wilmington, DE) at 37 ° C for 10 m and cooled to 4 ° C. The reaction was stopped with 2 μl 0.5 M EDTA and the RNA was immediately purified with the RNeasy MinElute Cleanup Kit (Qiagen, Valencia, CA), according to manufacturer instructions. All samples presented in Table 1 were subjected to globin depletion. Five samples were too dilute (<20 ng RNA/μL) for use in this protocol and were not subjected to globin depletion (Hua: June, Keo: July and September, Pele: April and June). RNA quality of globin depleted samples was assessed using an Agilent Bioanalyzer 2100 (Agilent Technologies, Inc., Santa Clara, CA).

### Reverse transcription and qPCR

To determine the extent of globin depletion and any non-specific impact on transcript levels, mRNA levels of HBA and four additional genes (ALAS2, FKBP8, GAPDH, RPL13) were assessed by quantitative real-time PCR in samples pre- and post-globin depletion. Fifty nanograms of RNA was reverse transcribed with EpiScript -RNase H Reverse Transcriptase (Epicentre, Madison, WI) and oligo(dT) priming. Gene specific primers (400 nM, Table 2) were used for qPCR on an ABI 7500 using ABI Power SYBR Green master mix (Applied Biosystems, Foster City, CA). The specificity of qPCR primers and the size of the amplicon were verified by analysis with an Agilent Bioanalyzer 2100 and further confirmed by melting curve analysis. The reaction efficiency was determined using a standard curve of cDNA from total RNA. A cycle threshold (Ct) was assigned at the beginning of the logarithmic phase of PCR amplification and the difference in the Ct values of the pre- and post-globin depletion samples were used to determine the relative expression of the gene in each sample.

**Table 2:** Primer sequences for qPCR analyses

Primer	Oligonucleotide Sequence (5’-3’)
HBA-F	ATGGACCGAGGGCGTGAAAT
HBA-R	GGTATTTGGAGGTCAGCACGG
ALAS2-F	TGATCCAAGGTATCCGCAATAG
ALAS2-R	GTGTCTCAGGGTTAGACTTCTTT
FKBP8-F	CCATCAAGGCCATCACTTCT
FKBP8-R	CCAGGTTGTTCAGACACTTCA
GAPDH-F	TATGACAACCACCTCAAGATCG
GAPDH-R	GCCGAAGTGGTCATGGATAA
RPL13-F	GTACCGCTCCAAGCTCATTCT
RPL13-R	CTCTGTGATGACTCTGGCTTTCT

### Sequencing

Globin depleted (*n* = 31, Table 1) and total RNA (*n* = 6; 5 samples named above and Kainalu May) samples were sent to North Carolina State University Genomics Service Laboratory for library preparation using a NEBNext Ultra Directional RNA Library Prep Kit for Illumina and indexed with the NEBNext Mulitplex Oligos for Illumina (New England Biolabs, Ipswich, MA). Sequencing was performed on an Illumina HiSeq 2500 sequencer (Illumina, San Diego, CA), at a targeted depth of 28 million, 100 nucleotide (nt) single end reads for globin depleted samples. Total RNA from samples that lacked sufficient RNA concentration to perform the globin depletion step were sequenced at a depth of 45 M reads, with the exception of Kainalu May which was sequenced at a depth of 28 M reads for a direct comparison with a globin depleted aliquot of the same sample.

### Genome-guided transcriptome assembly and analysis

Sequence processing and analysis was carried out in iPlant Collaborative’s Discovery Environment using the High-Performance Computing applications [15]. The Illumina BCL output files were converted to FASTQ-sanger file format and sequence quality trimming was performed using Trimmomatic [16], with a minimum phred quality score >20 over the length of the reads. The trimmed reads were then quality checked using the FASTQC tool. To assess the effectiveness of globin depletion, reads were mapped to the Ensembl *T. truncatus* genome, turTru1 v76.1, using Tophat2 v 2.3.13 [17] with Bowtie2 v 2.2.4 [18] as the alignment engine and mapped read counts, as FPKM (fragments per kilobase of transcript per million mapped reads), were generated using Cufflinks v 2.2.0 [19] with the genome as a reference. Differential expression analysis was performed using Cuffdiff v 2.1.1 [19] and visualization generated by CummeRbund [19]. For more detailed gene expression analysis of the blood transcriptome, reads from globin depleted samples were mapped to the Ensembl *T. truncatus* genome, turTru1 v76.1, using RSEM v 1.2.18 [20] with Bowtie2 v 2.2.4 [18] as the alignment engine and mapped read counts, as FPKM (fragments per kilobase of transcript per million mapped reads), were generated. Differential expression analyses were performed in EBSeq [21] using an FDR of 0.05. The raw reads and summarized FPKMs for all samples are available on GEO (accession # GSE78770). Gene enrichment analysis and pathway mapping of the differentially expressed gene sets was analyzed using Fishers Exact test in Blast2GO [22–25] (FDR < 0.05) and pathway mapping with the hypergeometric test for enrichment evaluation in WebGestalt [26, 27] (Benjamini & Hochberg adjusted *p*-value < 0.05) using a background comprised of all genes expressed in blood with an average FPKM ≥ 1 across all samples (*n* = 31) and and FPKM > 0 in at least half of the samples.

### *de novo* transcriptome assembly and analysis

The processed and trimmed reads were also used to construct a *de novo* transcriptome using the Trinity assembler [28] on iPlant Collaborative’s Discovery Environment. The read files from one summer and one winter globin depleted sample from each animal (*n* = 8; Hua: Feb and Sept, Kainalu: Feb and Aug, Keo: Feb and Aug, Pele: Feb and Sept) were concatenated into a single fastq file for assembly using a minimum K-mer coverage of 1, a minimum overlap value of 25 and a minimum contig length of 400 nucleotides. The assembly completeness was assessed by mapping a set of highly conserved core eukaryotic genes using CEGMA [29]. The transcriptome was annotated using BLAST+ for blastx searches (*E*-value ≤ 1e^−4^) of the human subset of the UniProt-SwissProt database (downloaded 10Jun2016), as the Ensembl genome is annotated off the human genome, followed by conserved domain mapping and gene ontology assignment using Blast2GO [22–25]. Read mapping, quantification, differential expression, gene enrichment analysis and pathway mapping were carried out using the same methods as in analysis of the genome-guided assembly. Gene enrichment and pathway mapping used a background comprised of all genes expressed in blood with an average FPKM ≥ 1 across all samples (*n* = 31) and FPKM > 0 in at least half of the samples. The Trinity assembly, raw reads, summarized FPKMs, and differential expression results are available on GEO (accession # GSE78770).

### Principal components analysis

Principal component analysis (PCA) was performed on log2 transformed FPKM values for all genes that had an FPKM > 0 in at least half of the samples and an average FPKM value across all samples of ≥ 1 FPKM. PCA was performed using the prcomp package from the stats library in RStudio (v 0.99.486). The plots were visualized using ggplot2 (v 1.0.1) [30].

### Cosinor analysis

Seasonal expression patterns of genes found to be differentially expressed between summer (July, August, and September) and winter (December and February) months were explored by transforming longitudinal data from all samples (*n* = 31) to estimate a cosinor linear model [31] using the cosinor package (v 1.1) [32] in Rstudio (v 0.99.878) and visualized with ggplot2 (v 2.1.0) [30].

### Weighted gene Co-expression network analysis

A gene co-expression network was generated using WGCNA (v 1.51) [33] in R (v 3.3.0) on log_2_ transformed FPKM values for all genes that had an FPKM > 0 in at least half of the samples and an average FPKM value across all samples of ≥ 1 FPKM. An unsigned co-expression network was then constructed on all pairwise Spearman correlations of gene expression. To weight highly correlated genes, correlation coefficients were then raised to a soft thresholding power (β) of 10, as determined by scale-free topology [34]. For network construction, a minimum module size of 80 was used with a detect cut height of 0.90 and a merge cut height of 0.25. The resulting modules were then tested for their association with sample traits by correlating module eigengenes (the first principal component of the module, representative of the gene expression profiles) with clinical measurements as described in [33]. Gene ontology and pathway enrichment analyses were then performed on individual gene co-expression modules compared to a background of all genes expressed in blood using the hypergeometric test in WebGestalt as previously described.

## Results and discussion

### Effect of globin depletion

**Fig. 1:**
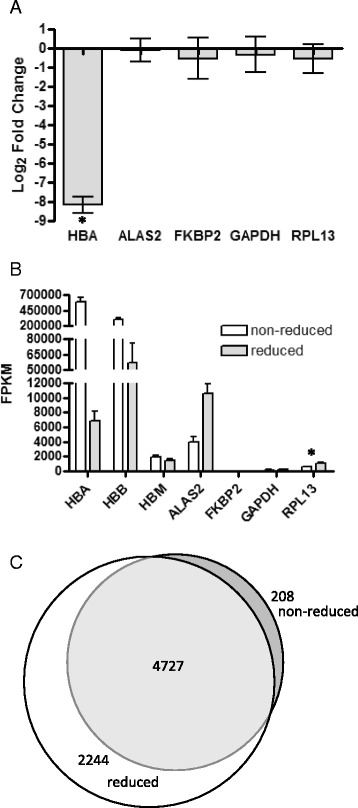
Globin depletion of dolphin blood RNA. **a** By real-time PCR, HBA was observed to be significantly depleted by nearly 300-fold (t-test, *p* < 0.05, *n* = 5), with little change in other genes (t-test, *p* > 0.05, *n* = 5). **b** Large reductions in HBA and HBB were observed by RNA-seq analysis in the absence of expression decreases in other genes (Cuffdiff, FDR < 0.05, *n* = 5). Due to extremely high expression values, statistical analyses were not performed on HBA and HBB. **c** Globin depletion of blood RNA resulted in a 10 % increase of identified genes. Statistical significance is denoted by an asterisk

As an analysis of a preliminary dolphin blood sample, sequenced at a targeted depth of 15 million reads, revealed that approximately 75 % of reads represented hemoglobin sequences (data not shown), we sought to establish a protocol for globin depletion of dolphin blood to increase the breadth of sequence detection. We modified an Affymetrix globin depletion protocol [12, 14] to yield high quality RNA with greatly depleted levels of hemoglobin transcripts. Recovery of RNA following globin depletion was high (median = 100.4 %) and only a minimal decrease in RIN, from 8.4 ± 0.06 to 7.8 ± 0.09, was observed. It has been reported that RNA recovery is low and variable following globin depletion in human and porcine blood [12, 35] and decreased RINs are also common [12, 36], thus it appears the globin depletion protocol performed exceptionally well in dolphin blood. As measured by quantitative PCR, HBA was significantly reduced by 286.4 ± 1.3 fold (t-test, *p* < 0.05, *n* = 5), whereas expression of other selected genes was not significantly changed (t-test, *p* > 0.05, *n* = 5) (Fig. 1a). Due to limitations in RNA quantity, only a single sample (Kainalu May) was sequenced at a targeted depth of 28 million reads both pre- and post-globin depletion. In this sample HBA was reduced 99.5 %, HBB 92 %, and HBM 35.8 %. To further investigate the effects of globin depletion in our study, five pairs of globin depleted and non-depleted samples, each collected 1 month apart, were analyzed by RNA-seq. HBA and HBB were reduced by 98.8 and 80.9 % respectively (Fig. 1b). This degree of depletion is similar to that observed using this protocol in porcine samples [12]. HBM exhibited a minor, non-significant decrease of 1.3 fold. In addition, ENSTTRG00000012084, annotated as a novel protein coding gene in the dolphin genome, was expressed at exceptionally high levels in non-depleted samples and was virtually undetected in globin depleted samples (99.9 % reduction). While this gene is termed “novel” in the genome annotation, it is located on a gene scaffold containing only hemoglobin genes (HBA, HBM, HBQ1, and HBZ). Blastx searches of the NCBI nr database identify this gene as HBA (*E*-value = 4e^−37^). Likewise ENSTTRG00000009506, annotated as a novel protein coding gene in the Ensembl genome, was reduced by 71.6 % following globin depletion. This gene is located on a scaffold with HBB and HBE and blastx searches identified this gene as HBB (*E*-value = 4e^−98^). Despite large reductions in expression, this gene and the annotated HBB were the top two most highly expressed genes in globin depleted samples, indicating that further protocol modifications may improve the reduction of HBB. Overall, globin depletion had little impact on expression of other genes, with only 790 (3.7 %) genes showing significantly different expression between depleted and non-depleted samples (Cuffdiff, FDR < 0.05, *n* = 5). Among these, 357 exhibited 1.6–13.9 fold higher expression in non-depleted samples, while 433 had 1.6–18.4 fold higher expression in globin depleted samples. As these differences may encompass both biological and technical variation, all further analyses were conducted on the globin depleted samples only.

As a result of globin depletion, 2244 additional genes (10.6 % of genome) were detected at a FPKM ≥ 1 (Fig. 1c), similar to the 8.6 % or 9.8 % increase in gene detection following globin depletion of porcine [12] or human [37] blood, respectively. Due to limiting amounts of starting RNA, five samples were not subjected to globin depletion and were instead sequenced at a targeted depth of 45 million reads. These samples were compared to five temporally matched globin depleted samples sequenced at a targeted depth of 28 million reads. Only 268 additional genes (1.3 % of genome) were detected with an average FPKM ≥ 1 in globin depleted samples. Thus, it is likely that globin depletion of dolphin blood is not necessary for RNA-seq studies if sequenced to a sufficient depth to overcome high levels of globin expression, as has been reported for human blood [37]. Our study suggests that approximately 45 M reads is sufficient to overcome globin dominance of the transcript pool.

### Genome-guided assembly transcript expression in blood

**Fig. 2:**
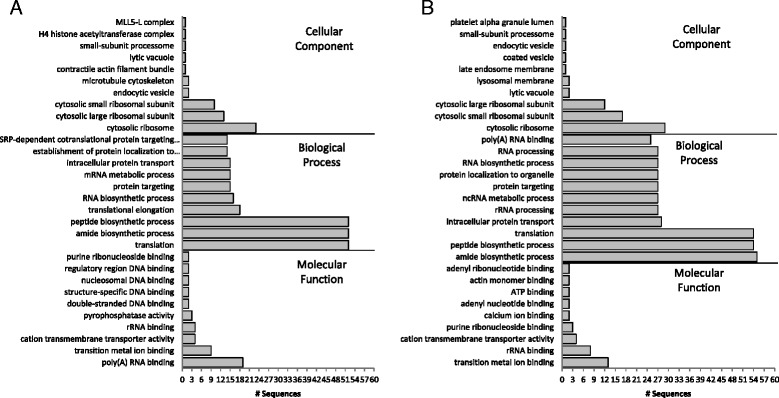
Top ten Gene Ontology (GO) annotations (level 6) from the 100 most highly expressed transcripts in dolphin blood. **a** All of the top 100 expressed genes mapping to the dolphin genome were annotated in Blast2GO. **b** Ninety-two of the top 100 expressed genes mapping to the *de novo* blood transcriptome were annotated in Blast2GO

Overall approximately 85 % of reads mapped back to the dolphin genome. However, only 28.5 % of reads mapped back to annotated genes in the genome with Bowtie2. This indicated that many reads mapped outside of annotated regions of the genome and were consequently excluded from further analyses; therefore improved annotation of the Ensembl dolphin genome may greatly expand data interpretation. In order to more accurately compare the dolphin blood transcriptome to the Ensembl genome, we selected a suite of 17,475 sequences, comprised of coding sequences and pseudogenes, from the genome. Pseudogenes were included after identifying reads from the transcriptome aligning to regions of the genome annotated as such. Reads mapped to 9610, 45.2 %, of these genes identified in the Ensembl dolphin genome, with FPKM > 0 in at least half the samples and an average FPKM ≥ 1, similar to the percentage expressed in human blood [35]. Among the 100 most highly expressed genes in dolphin blood (avg FPKM from globin depleted samples), 100 % were annotated and were dominated by transcripts associated with ribosomes, translation, and DNA and RNA binding (Fig. 2a). Many of these terms are also among the most highly expressed transcripts in human blood. Likewise, transcripts mapping to GO terms involved with immune response, transcription, cell cycle and proliferation, signaling, and structural components or functions are highly expressed in both human and dolphin blood [38].

Pathway mapping indicates that many basic cellular functions and processes, and in particular immune functions, are well represented within the blood transcriptome (Table 3). In all, WebGestalt identified 137 pathways (KEGG or WikiPathway) significantly enriched in the blood transcriptome relative to the genome (Benjamini-Hochberg *p*-value < 0.05). In contrast, the 7865 genes not expressed in blood only showed enrichment of 24 different pathways, notably lacking many basic metabolic functions and processes encompassed by blood transcripts (data not shown). Similarly, an analysis of GO terms found the blood transcriptome to be significantly enriched in processes and functions associated with the ribosome, transcription, translation, cell cycle, protein processing, cellular homeostasis, and abiotic and immune responses (Fisher’s exact test, FDR < 0.05). Many GO terms related to tissue-specific processes and functions are not expressed in blood including sensory processes (olfactory, visual, taste) and skeletal and cardiovascular system development (Fisher’s exact test, FDR < 0.05). Hormone-related GO terms have been documented in the human blood transcriptome [2, 39], however GO terms for hormone activity and signaling and synaptic functions were significantly under-represented in the dolphin blood transcriptome. Only 3 of 47 genes mapping to hormone activity (GO:0005179), hormone-mediated signaling pathway (GO:0009755), or hormone metabolic process (GO:0042445) were present in the blood transcriptome. As data sets were queried to ensure that sex-specific expression was not causing these transcripts to be excluded from our analysis (i.e., FPKM > 0 was required in at least half of samples) it is unknown why a broad representation of hormone-related transcripts are not present in the dolphin blood transcriptome, but it is possible that hormone-related transcripts are poorly annotated in the current genome.

**Table 3:** Pathways of interest significantly represented in the blood transcriptome

Pathway	Pathway #	#T/#G	*p*-value
Protein processing in endoplasmic reticulum	K4141	136/149	5.58E-17
Metabolic pathways	K1100	663/916	8.49E-17
mRNA processing	WP411	108/116	1.51E-14
Lysosome	K4142	102/111	2.62E-13
Ubiquitin mediated proteolysis	K4120	114/128	1.59E-12
Ribosome	K3010	68/70	2.93E-12
Electron Transport Chain	WP111	67/69	1.08E-11
Apoptosis	K4210	70/75	4.72E-10
Oxidative phosphorylation	K190	82/92	3.15E-09
B cell receptor signaling pathway	K4662	63/68	7.37E-09
T cell receptor signaling pathway	K4660	85/99	7.68E-08
Neurotrophin signaling pathway	K4722	97/116	1.11E-07
Toll-like receptor signaling pathway	K4620	66/75	3.91E-07
Natural killer cell mediated cytotoxicity	K4650	71/83	1.41E-06
Translation Factors	WP107	41/43	2.56E-06
Androgen receptor signaling pathway	WP138	73/86	6.13E-06
Antigen processing and presentation	K4612	34/36	1.45E-05
DNA replication	K3030	31/33	5.79E-05
MAPK signaling pathway	WP382	117/153	8.20E-05
NOD-like receptor signaling pathway	K4621	42/48	1.00E-04
Proteasome	K3050	38/43	2.00E-04
Phagosome	K4145	84/109	3.00E-04
RIG-I-like receptor signaling pathway	K4622	45/53	3.00E-04
Citrate cycle (TCA cycle)	K20	26/28	4.00E-04
Endocytosis	K4144	127/175	5.00E-04
Protein export	K3060	21/22	5.00E-04
Insulin signaling pathway	K4910	92/123	8.00E-04
Peroxisome	K4146	56/72	2.30E-03
Primary immunodeficiency	K5340	28/33	4.60E-03
IL-7 signaling pathway	WP205	22/24	5.60E-03
Chemokine signaling pathway	K4062	105/152	1.82E-02

WebGestalt analysis using an Benjamini Hochberg adjusted *p*-value. The genome (G) used for background contained 17,475 coding sequences and psuedogenes. The test (T) set was the 9610 genes expressed in blood. *WP* WikiPathways, *K* KEGG pathway

### *de novo* transcriptome-guided assembly transcript expression in blood

A Trinity assembly of reads combined from eight samples resulted in 49,925 contigs with a minimum length of 400 nucleotides. The *de novo* assembly had an N50 of 1331 nt and the longest contig was 12,295 nt in length. The assembly appears to encompass the breadth of core eukaryotic genes (CEGs), with 87.5 % of full length CEGs identified by CEGMA. This increases to 97.78 % of CEGs when partial-length alignments to CEGs are included. When the *de novo* transcripts were aligned to the coding subset of the genome via blastn, 31.5 % of transcripts returned hits with an *E*-value < 1e^−4^, notably similar to the percentage of reads mapping back to annotated transcripts in the genome-based analysis and indicating a number of unannotated coding sequences in the Ensembl genome or novel sequence information identified by the *de novo* transcriptome assembly. However, blastn alignments of the *de novo* transcriptome to the full genome sequence indicate that there are minimal novel sequences in the *de novo* assembly. Rather, many transcripts in the *de novo* assembly map outside of annotated regions of the Ensembl dolphin genome; therefore the reduced mapping to the genome likely reflects the limited annotation available rather than an absence of sequence data. Overall, 88 % of reads mapped back to the transcriptome using Bowtie2. This is significantly higher than the 28.5 % observed mapping to annotated genes in the genome, yielding a substantial increase in usable read data for downstream analyses (Wilcoxon signed rank test, *p* < 0.0001). Thus, all further analyses presented herein utilize the *de novo* assembly.

When filtered to ensure FPKM > 0 in at least half of samples and an average FPKM ≥ 1 there were 29,702 transcripts (59.5 % of *de novo* transcriptome) and 13,889 had homology to human genes via blastx searches as described in the methods, a sizable increase from the 9610 (45.2 % of genome) genes mapping to the genome. Thirty-eight percent of these transcripts were fully annotated in Blast2GO and 21 % mapped unambiguously to Entrez Gene IDs for pathway mapping in WebGestalt. This set of 29,702 transcripts was defined as the “blood transcriptome” and used as the background set of transcripts expressed in blood for all further analyses. There was no significant enrichment of any GO terms among this background set relative to the complete *de novo* assembly, likely reflecting the expected breadth of gene expression in the blood transcriptome. Overall, gene expression guided with the *de novo* transcriptome was similar to that guided by the dolphin genome. Among the top 100 most highly expressed genes in the transcriptome (avg FPKM from globin depleted samples), 92 % were annotated, and were likewise dominated by transcripts associated with the ribosome, protein and nucleic acid binding, and translation (Fig. 2b). As in the genome-based analysis, there appeared to be little expression of genes involved in hormone biosynthesis, degradation, and signaling in the blood transcriptome, with only 12 transcripts mapping to hormone activity (GO:0005179), hormone-mediated signaling pathway (GO:0009755), or hormone metabolic process (GO:0042445) in the blood transcriptome. KEGG or WikiPathway analysis in WebGestalt identified significant mapping to ribosome pathways (*p*-value < 5e^−70^). The absence of hormone related transcripts in dolphin blood may reflect differences in hormone related transcript expression between humans and dolphins or may be due to the lack of homology, at the sequence level, between human and dolphin transcripts.

### Overall variation between samples using principal components analysis

**Fig. 3:**
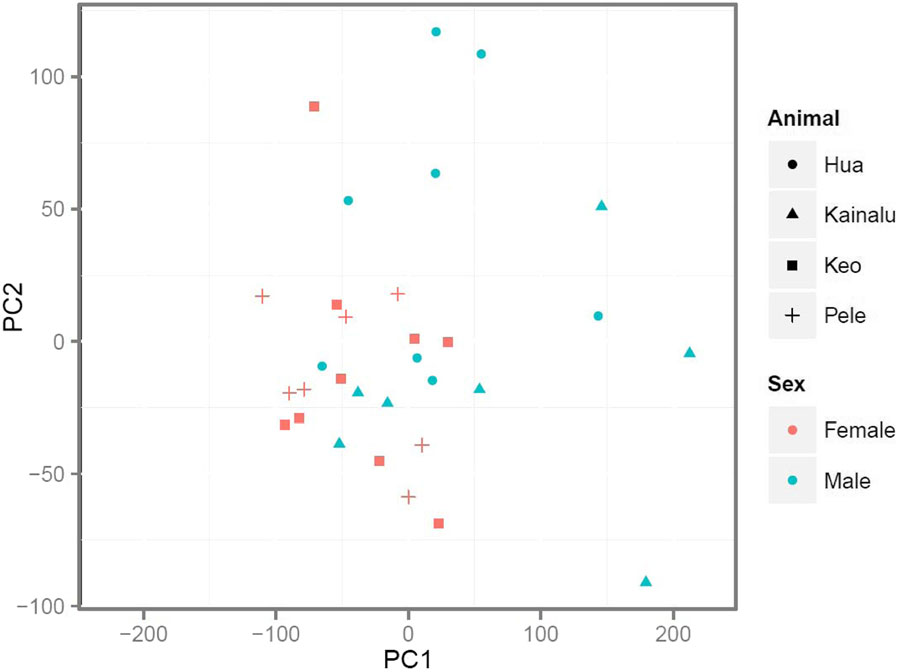
Principal components analysis of 31 transcript profiles. The transcript profiles from the females (*pink*) largely clustered together, while those from the males (*blue*) were much more variable. Although the profiles from both males were variable, the young male (Hua, 5 years) differed somewhat from those of the older male (Keo, 17 years) along PC2

Principal components analysis did not reveal strong clustering associated with animal, sex, season, or any other measured parameter. PC1 accounted for 21.8 % of the variance and was somewhat correlated with sex, with females clustering together while the expression profiles from the two males were more variable (Fig. 3). PC2 only accounted for 7.8 % of variance and was not associated with animal, sex, or season. Samples from individual animals did not cluster together on either the PC1 or PC2 axes. The samples from males that clustered with the females on PC1 were not consistent with regard to season of collection. Neither PC1 nor PC2 were correlated with day length, water temperature, month or season of collection, nor with any of the hematological parameters measured.

### Genes expressed differentially by sex

Of the 29,702 expressed genes the blood transcriptome, 499 (1.7 %) were differentially expressed (EBseq, FDR <0.05) between males (*n* = 15, all samples from Hua and Kainalu) and females (*n* = 16, all samples from Keo and Pele) (Additional file 2: Table S2). Of these, 204 (41 %) had annotation. Two were homologs to X chromosome-linked genes in humans: SMC1A and CD40LG. Both were expressed slightly higher in females (log_2_ fold change 0.45). SMC1A, required for sister chromatid cohesion during cell division is known to be located in an area of the X chromosome that is not subject to X inactivation [40]. No homologs to human Y chromosome genes were found among the differentially expressed transcripts. Interestingly, while most dolphin chromosomes display substantial homology to human chromosomes by chromosome painting, the Y chromosome in dolphins is minute, and does not display any cross-hybridization with human chromosome probes [41]. Thus the absence of Y chromosome genes anticipated in sex biased gene expression may reflect a lack of homology to human genes, resulting in unannotated dolphin transcripts. Ninety-three genes were essentially expressed only in males (female ave FPKM < 1). Of these 11 were annotated: BATF1, CENPF, CREG1, GALM, HSF1, NDRG3, NOXO1, RAB22A, SLC18A2, SPAST, and TSLNG and all are autosomal in humans. Overall, 261 genes were expressed more highly in males (log_2_ fold change 0.28 to 11.9, ave = 1.65). Among the 238 genes more highly expressed in females (log_2_ fold change 0.28 to 10.4, ave = 1.84), 151 were essentially unexpressed in males (male ave FPKM < 1). The ten annotated female expressed genes, C20orf112, CHMP1A, CNN2, PPP4R2, SAMHD1, SNX19, TXLNG, ULK1, VPS13C, ZDHHC21, are all autosomal in humans. All other genes in the differentially expressed list were present in both sexes, but at different levels. This sex-biased gene set had ten genes in common (among those annotated) with those differentially expressed in human peripheral blood, which included 582 autosomal genes [42], plus 51 X-chromosome linked and 26 Y-chromosome linked genes. In humans the gene ontology processes enriched in the female biased genes included cytokine stimulus, response to interferon, and lymphocyte differentiation, while male specific genes were not enriched for any Biological Process GO category. There was no enrichment of any GO term among the annotated genes expressed more highly in male or female dolphins, nor in the combined set of genes differentially expressed by sex in this study.

### Seasonal changes in gene expression

As significant seasonal changes in transcript expression have been observed in human blood [11] and dolphin skin [43], we queried this data set for transcripts differentially expressed between summer and winter. Based on local temperatures, samples from July, August, and September were collectively defined as summer (*n* = 8) while samples from December and February were defined as winter (*n* = 8). EBSeq reported a very small percentage of the dolphin blood transcriptome exhibiting significant changes in expression between summer and winter months (Additional file 3: Table S3). Overall, 0.7 % of the transcriptome exhibited significant changes between summer and winter months (FDR < 0.05, log_2_ fold change −4.9 to 4.2) and only 53 were annotated. The majority of genes, 63.8 %, were more highly expressed in summer (ave log_2_ fold change 2.18). The 36.2 % of genes more highly expressed in winter exhibited an average log_2_ fold change of 2.4. Fifty-three were effectively expressed only in winter (summer ave FPKM < 1), whereas 102 were expressed only in summer (winter ave FPKM < 1). There is no significant enrichment of any GO category or pathway in these sets of seasonally changing transcripts.

Despite the minimal changes in gene expression correlated with sex, seasonal gene expression was queried for males and females separately. Among males only a similar amount of change was observed, 0.8 % of transcriptome, whereas 2.4 % of the transcriptome changed significantly in females with season (FDR < 0.05, Additional file 3: Table S3). These gene sets are distinct, with only seven genes in common between the male and female sets of seasonal changers. Only one of the seven genes was annotated; highly similar to human beta-chimaerin. While there was no significant enrichment of any GO terms or pathways in these data sets, not only did the female only analysis yield the largest number of differentially expressed genes, it also exhibited the greatest degree of change (log_2_ fold change −6.86 to 6.55).

#### Similarities with human seasonal gene expression

The percentage of genes exhibiting seasonal change in dolphin blood (approx. 0.7–2.4 %) is markedly less than the 23 % observed in human blood [11] or 25 % in dolphin skin [43]. This may be due to the minimal seasonal fluctuations in temperature (1.5 ° C) or day length (1.5 h) prevailing in the Waikoloa, Hawaii area, and may also reflect the small sample set in the current study (i.e., larger variance). Nonetheless, similarities exist among the gene sets, with 16 % of seasonally expressed genes assigned a gene symbol via blast searches in dolphin blood also present in the list of seasonal genes from human blood. An additional 42 % of annotated seasonal genes in dolphin blood are represented by a different member of the same protein family in human blood. This trend was more apparent in the slightly larger male-only seasonal gene set, with 40 % of annotated genes also found in the human seasonal set and another 40 % represented by another member within the protein family in human blood. The largest seasonal gene set from the female-only analysis had substantial overlap with the human set (47 % same gene, 32 % same family). In contrast, there was much less overlap with a recent study identifying seasonal change in gene expression in dolphin skin from the Gulf of Mexico. Fewer than 5 % of seasonal genes in dolphin blood are the same as seasonal genes identified in dolphin skin via microarray and approximately 33 % are represented by another gene in the same family [43]. The difference between the dolphin blood and skin transcriptomes may reflects tissue specific differences in gene expression as well as differences in environmental exposures of the two tissue compartments to temperature fluctuations. Further, the blood transcriptome utilized samples from managed dolphins in Waikoloa, Hawaii whereas the skin transcriptome utilized samples from wild dolphins in the northern Gulf of Mexico.

**Fig. 4:**
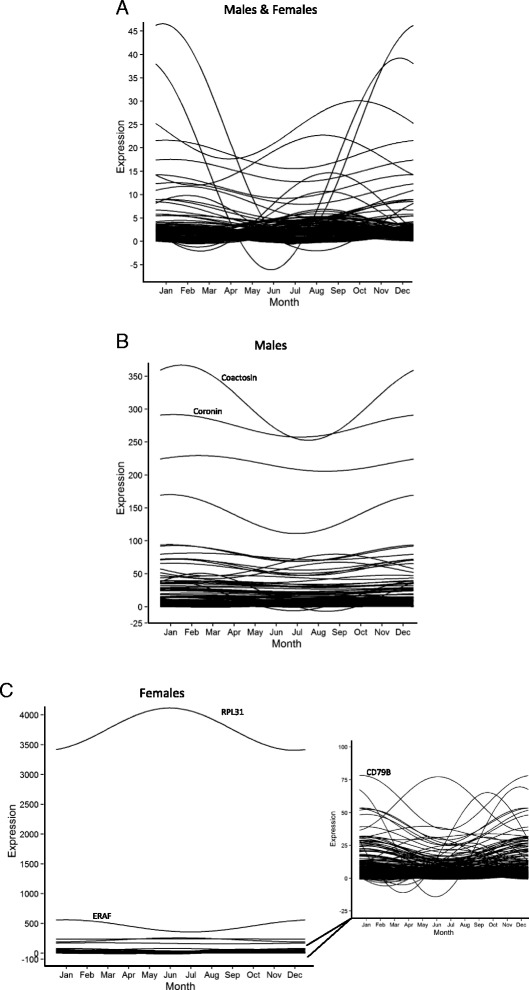
Cosinor analysis of seasonally-expressed genes in the dolphin blood transcriptome. Genes differentially expressed between summer (July, August, September) and winter months (December and February) (EBseq FDR < 0.05) are plotted for males and female (**a**, *n* = 31, 210 transcripts), in males only (**b**, *n* = 15, 252 transcripts), or in females only (**c**, *n* = 16, 699 transcripts). Expression profiles are varied and there is little overlap between the three gene sets

While the seasonal impacts on gene expression may be muted by the tropical climate or small sample size in this study, the agreement observed between the dolphin blood and human blood data sets indicates that gene expression in dolphin blood may undergo seasonal variation that must be taken into account when assessing gene expression changes associated with clinical parameters, disease, or toxic exposures. To this end, genes that significantly differed in expression between summer and winter months were subjected to a cosinor analysis to visualize any seasonal expression cycles. Overall, genes differentially expressed by season exhibited peak expression either in cooler months (November – February) or in warmer months, (June - September), however patterns and extent of cyclic changes in expression varied between data sets (Fig. 4). Among the genes differentially expressed when analysis was performed on the full data set (males and females), the two transcripts with the highest expression levels and greatest degree of change exhibited peak expression during cooler months, but both are unannotated (Fig. 4a). Analysis of the differentially expressed genes in males only exhibited more stable annual expression patterns (Fig. 4b). The two most highly expressed genes, which exhibited peak expression in cooler months, are coactosin and coronin, both associated with cytoskeletal processes and actin binding. Differentially expressed genes in females only again exhibited the greatest annual rhythmicity among the analyzed data sets (Fig. 4c). A 60S ribosomal protein L31 (RPL31) has the highest expression and greatest amplitude of change, with peak expression in cooler months. An erythroid associated factor (ERAF) exhibited high expression levels, peaking in warmer months, whereas CD79B exhibited seasonal rhythmicity peaking in cooler months. These seasonal expression changes in ERAF and CD79B may reflect changes in the cellular composition of blood [10, 11]. A larger sample set, as well as samples from dolphins in regions undergoing greater environmental fluctuations over the year may reveal cyclic patterns of gene expression not observed in the current study.

### Gene co-expression network analysis

**Fig. 5:**
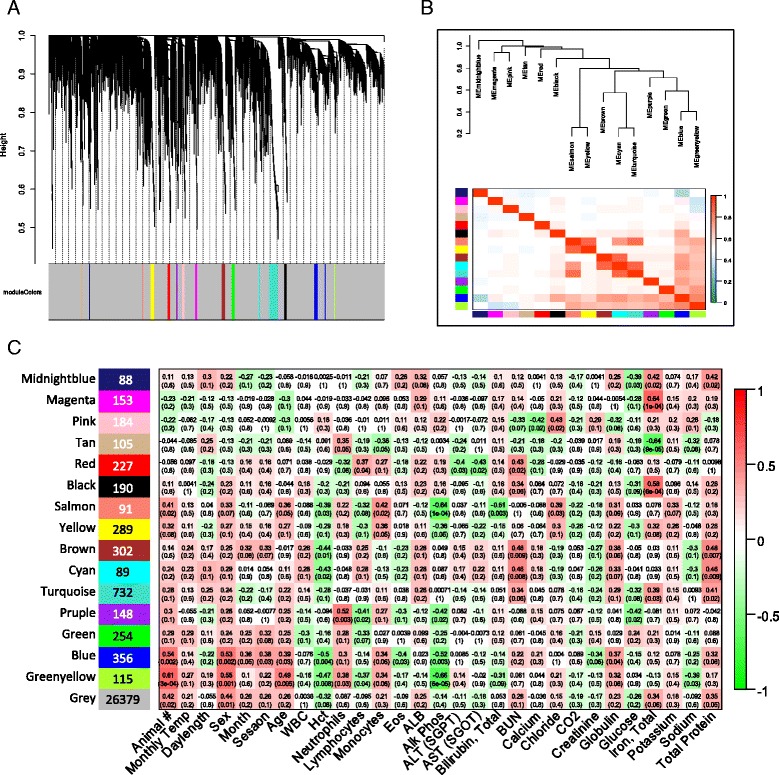
Weighted gene expression co-variance network analysis (WGCNA) identified 15 co-expressed gene modules. **a** Average link hierarchical clustering dendrogram of the network with color bands identifying module membership. **b** Hierarchical cluster of module eigengenes identifies closely related modules. **c** Correlation matrix of modules with sample traits and hematological parameters: *red* is positively correlated, *green* is negatively correlated. The correlation coefficient between the module eigengene and the measured trait is listed for each pairwise correlation, with significance in parentheses (*p*-value). Number of genes in each module is listed at left

The utility of the blood transcriptome to identify physiological perturbations, such as those resulting from disease or toxic exposure, requires insight into the stability of the healthy transcriptome over time, as well as differences between individuals that may relate to differences in age, sex, or hematological parameters. We therefore constructed a gene co-expression network in WGCNA using all samples (*n* = 31) as independent measures. Fifteen co-expressed gene modules were identified (Fig. 5a and b), while the majority of genes, represented by the grey module, were not significantly co-regulated. Gene membership in these modules is listed in Additional file 4: Table S4. Pairwise correlations between each module eigengene and each of the physical or hematological parameters measured revealed several modules with significant associations (Fig. 5c). There were no co-expressed gene modules associated with temperature or day length, and only modest correlation to season (blue module, *r* = 0.38, *p* = 0.04). Only two modules showed strong correlation to individual animal, the blue module (352 transcripts, *r* = 0.54, *p* = 2e^−03^) and the greenyellow module (115 transcripts, *p* = 0.61, *p* = 3e^−04^). These modules also correlated significantly with the sex and age, and were negatively correlated with hematocrit and alkaline phosphatase. Hematocrit values were significantly different between sexes (mean ± SEM = 38.7 ± 0.49 in females; 43.75 ± 0.53 in males). The correlation with alkaline phosphatase likely reflects the high alkaline phosphatase levels observed in the young male (Hua, 5 years; range 570–848 U/L) and the low alkaline phosphatase levels found in the older female (Pele, 28 years; range 107–190 U/L). Alkaline phosphatase levels have previously been shown to be high in juvenile dolphins [44]. The KEGG pathway for map kinase signaling (*p* = 0.015) was enriched in the blue module while KEGG pathways for hematopoeitic cell lineage (*p* = 7.2^e-03^) and regulation of the actin cytoskeleton (*p* = 7.2^e-03^) were enriched in the yellowgreen module. The salmon module, although more weakly associated with sex and age, was also strongly associated with alkaline phosphatase (*r* = −0.68, *p* = 2e^−05^), however the small number of annotated transcripts in this module prevented the identification of any enrichment within the module.

The brown module (302 genes) had little correlation with animal, sex or age, but was negatively correlated with hematocrit and positively correlated with blood urea nitrogen (BUN), a proxy for kidney function, and total protein. The brown module was enriched in GO terms for regulation of muscle fiber development (*p* = 9.7e^−03^) and endothelial cushion morphogenesis (*p* = 6e^−03^), and KEGG pathways for gluconeogenesis (*p* = 6.0e^−03^), galactose and starch metabolism (*p* = 2.7e^−02^), and gluconeogenesis (*p* = 3.6e^−02^). The cyan module (89 genes) has similar correlations to hematocrit and BUN but had no significant enrichment, probably due to its small size.

The tan module (105 genes) was negatively correlated with total iron (*r* = −0.64, *p* = 9e^−5^), and enriched in GO terms for circulatory system process (*p* = 1.2e^−02^) and blood circulatory genes (*p* = 1.2e^−02^). Two other modules (magenta and black) with positive correlation to total iron had no significant enrichment.

The purple module (145 genes) was positively correlated with neutrophils (*r* = 0.52, *p* = 0.003) and negatively correlated with lymphocyte percentage (*r* = −0.41, *p* = 0.02), alkaline phosphatase (*r* = −0.42, *p* = 0.02), and glucose (*r* = −0.42, *p* = 0.02). There was no significant difference in lymphocyte percentage between the sexes; however, the 17 year old male, Kainalu, was substantially lower in lymphocyte percentage (13.25 ± 3.0) than all other animals (20.9 ± 5.7) and was also below the normal range for dolphins (15–30 %). This module was enriched in GO process categories for negative regulation of sequestration of calcium (*p* = 3.2e^−02^) and cell cycle (*p* = 2.3e^−02^).

The ability of the network analysis to identify modules of co-expressed genes that correlate with clinical measurements in this small sample of healthy, managed dolphins suggests that blood transcriptomes may be informative for identifying metabolic perturbations indicative of with infections, disease, or toxic exposures in bottlenose dolphins. The independence of many co-expressed modules from the individual animal and month of collection suggests that these may not be confounding factors for identifying transcriptomic responses to adverse health impacts.

## Conclusions

This longitudinal analysis of blood transcriptomes from four managed bottlenose dolphins provides the first information on the blood transcriptome content and sex, seasonal, and individual variation in transcript expression in bottlenose dolphins. The blood transcriptome was found to express a wide array of genes that mapped to diverse pathways, thus demonstrating the potential for broad applications of dolphin blood transcriptomic analysis in marine mammal management. We found both a seasonal component to changes in blood gene expression, consistent with studies in humans, and an association of gene co-expression modules with age, sex or hematological parameters measured. However, the proportion of genes exhibiting changes in expression along with the degree of change observed was limited, demonstrating the relative stability of the dolphin blood transcriptome within and between animals throughout the course of a year. Although this represents a small sample of healthy, managed dolphins, the observed correlations to hematological parameters coinciding with an otherwise stable transcriptome and precedence from human medicine suggests that blood transcriptome analysis may be useful for identifying exposures, infections, and pathological changes that cannot be readily monitored in protected marine mammal species. The utility of blood transcriptomics for diagnostic purposes in bottlenose dolphins will require the establishment of a robust database of gene expression in dolphins from different environments, both managed and wild, with which to establish normative values in healthy animals. The establishment of such an archive would facilitate the use of blood transcriptomics for biomonitoring in wild and managed populations of bottlenose dolphins.

## Additional files

Additional file 1: Table S1. All globin depleted samples and associated physical or hematological parameters measured. (XLS 43 kb) Additional file 2: Table S2. Genes with significantly different expression between males and females (EBSeq, FDR < 0.05). (XLS 85 kb) Additional file 3: Table S3. Annotated genes with significantly different expression between summer and winter from de novo transcriptome based analyses (EBseq, FDR < 0.05). (XLS 166 kb) Additional file 4: Table S4. Gene co-expression modules with significant correlation to measured traits. (XLS 427 kb)

## Abbreviations

AMMPA: Alliance of marine mammal parks and aquariums
BAR: Bright, alert responsive
BLAST: Basic local alignment search tool
CEG: Core eukaryotic gene
CEGMA: Core eukaryotic genes mapping approach
FPKM: Fragments per kilobase of transcript per million mapped reads
GEO: Gene expression omnibus
GO: Gene ontology
PC: Principal component
PCA: Principal component analysis
PCB: Polychlorinated biphenyl
PCR: Polymerase chain reaction
RIN: RNA integrity number
RSEM: RNA-seq by expectation maximization
WGCNA: Weighted gene co-expression network analysis
